# Fragilidade e Fibrilação Atrial: Um Olhar Mais Atento ao Estudo FRAIL-AF

**DOI:** 10.36660/abc.20230671

**Published:** 2024-06-03

**Authors:** Roberto Muniz Ferreira

**Affiliations:** 1 Universidade Federal do Rio de Janeiro Instituto do Coração Edson Saad Rio de Janeiro RJ Brasil Universidade Federal do Rio de Janeiro, Instituto do Coração Edson Saad, Rio de Janeiro, RJ – Brasil; 2 Hospital Samaritano Departamento de Cardiologia Rio de Janeiro RJ Brasil Hospital Samaritano – Departamento de Cardiologia, Rio de Janeiro, RJ – Brasil

**Keywords:** Fibrilação Atrial, Anticoagulantes, Medicina Baseada em Evidências, Idosos Fragilizados

## Introdução

Embora a medicina baseada em evidências (MBE) tenha contribuído para estabelecer as bases do atendimento ao paciente há mais de 40 anos, os princípios científicos ainda são frequentemente desconsiderados na tomada de decisões clínicas. Como resultado, a MBE pode ser erroneamente caracterizada como uma barreira que limita a aplicabilidade de conceitos pouco reconhecidos, de outra forma percebidos como totalmente apoiados pela literatura médica. A análise crítica das publicações médicas não deve basear-se em métodos alheios ao domínio científico, e a MBE fornece os meios para limitar tais equívocos.

Apesar do progresso contínuo no conhecimento médico, numerosas áreas de incerteza persistem na prática clínica. Nesses casos, a tomada de decisões depende normalmente de informações provenientes de cenários diferentes, de investigações ou experiências pessoais concebidas de forma inadequada e de alternativas que são inerentemente frágeis como provas de embasamento. Na verdade, a fragilidade é um problema que a maioria dos médicos, em última análise, tem de gerir, seja no que diz respeito às evidências científicas ou aos perfis dos pacientes. Maiores dificuldades surgem quando ambos são uma fonte de fragilidade, uma vez que decisões mal avaliadas tendem a ter resultados ainda piores. A fibrilação atrial (FA) é um tema onde essa associação potencialmente ocorre.

## O ensaio FRAIL-AF

O estudo FRAIL-AF recentemente publicado (Segurança na troca de um antagonista da vitamina K para um anticoagulante oral não antagonista da vitamina K em pacientes idosos frágeis com FA) foi um ensaio de superioridade pragmático, aberto e randomizado que avaliou se pacientes frágeis com FA adequadamente tratada com antagonistas da vitamina K (AVKs) deveriam ser remanejados para o uso de anticoagulantes orais diretos (NOAC). Os principais critérios de inclusão consistiram em pacientes com idade ≥75 anos com pontuação do Groningen Frailty Indicator (GFI) ≥3, enquanto aqueles com FA valvular ou disfunção renal grave foram excluídos. O desfecho primário foi a primeira ocorrência de sangramento maior ou não maior clinicamente relevante (CRNM) ao longo de 12 meses de acompanhamento.^1^

Entre janeiro de 2018 e junho de 2022, 1.330 pacientes foram randomizados. A média de idade foi de 83 anos, a mediana do GFI foi de 4 e a maioria era do sexo masculino (61,2%). No grupo NOAC, a rivaroxabana foi a mais prescrita (50,2%), seguida da apixabana (17,4%) e da edoxabana (16,5%). Após um acompanhamento médio de 344 dias e 163 eventos de desfecho primário, o protocolo previamente definido exigiu a interrupção do estudo por futilidade, embora o grupo NOAC tenha sido associado a um aumento significativo de 69% nas taxas de sangramento (HR 1,69; IC 95% 1,23- 2,32; p=0,00112). Não houve diferenças em eventos tromboembólicos ou mortes.^1^

Com base nos dados disponíveis de vários ensaios randomizados e meta-análises que demonstram um perfil de segurança favorável dos NOACs quando comparados com AVKs, estes medicamentos foram estabelecidos como o regime de tratamento primário para a prevenção de eventos tromboembólicos para a maioria dos pacientes com FA. Grande parte do benefício deriva de reduções significativas em hemorragias graves, particularmente hemorragia intracraniana (HIC).^2 , 3^ Consequentemente, a *European Rhythm Association* (ERA) sugere que a anticoagulação não deve ser suspensa em indivíduos idosos com FA puramente com base na idade e que os NOACs também são a opção preferida nesta população.^3^

No entanto, o guia prático recentemente publicado sobre a utilização de NOAC desenvolvido pela ERA também reconheceu que a fragilidade caracteriza um grupo único de pacientes onde a indicação de qualquer anticoagulante oral ainda representa uma área de incerteza.^3^ A este respeito, Joosten et al.,^1^ devem ser elogiados por tentarem responder a uma das preocupações não abordadas adequadamente em grandes ensaios anteriores de NOACs.^1 , 4^ A fragilidade é uma síndrome que se estende além da idade avançada e envolve múltiplos fatores integradores, como polifarmácia e comorbidades graves, que acabam por impor um alto nível de dependência e vulnerabilidade. Na publicação acima mencionada, a ERA forneceu informações valiosas sobre a importância de uma avaliação geriátrica abrangente antes de a anticoagulação ser considerada neste contexto. Embora os autores tenham sugerido a Escala de Fragilidade Clínica do Estudo Canadense de Saúde e Envelhecimento (CHSA) para esse fim, outras escalas validadas também poderiam ser empregadas, como a GFI que foi utilizada no FRAIL-AF.^3 , 5 , 6^

A ERA recomendou que os indivíduos com FA classificados como gravemente frágeis pela escala CHSA normalmente não deveriam receber anticoagulação. Esses pacientes também seriam definidos como tendo uma pontuação GFI comparável de 3 (capazes de autocuidado limitado, confinados à cama ou cadeira e cerca de <50% das horas de vigília) ou 4 (completamente incapacitados, incapazes de realizar qualquer autocuidado, totalmente confinado à cama ou cadeira).^5 , 6^ Mais importante ainda, mais de 74% dos pacientes no FRAIL-AF tiveram uma pontuação GFI de 4 e, portanto, geralmente não seriam considerados para anticoagulação na maioria dos cenários clínicos, conforme proposto pela ERA.^1 , 3^ Esse perfil distinto do paciente ressalta uma das principais diferenças entre os estudos anteriores e o FRAIL-AF.

Uma razão fundamental para a exclusão sistemática de indivíduos frágeis de estudos anteriores é a baixa taxa de sobrevivência a curto prazo associada a essa condição. A maioria dos estudos sobre FA considerou valores mínimos de expectativa de vida de 1 a 2 anos para inclusão, limitando assim o número de pacientes frágeis randomizados.^4^ No FRAIL-AF, a taxa de mortalidade anual estimada no grupo AVK foi de 7,4%, o que foi aproximadamente 25% maior do que o subgrupo de pacientes ≥75 anos tratados com varfarina (5,97%) no estudo ARISTOTLE.^1 , 7^ Essa diferença nas taxas de sobrevivência enfatiza o efeito independente que a fragilidade tem no prognóstico, mesmo entre indivíduos idosos.

Além disso, o FRAIL-AF analisou um subgrupo particular de pacientes frágeis com FA, especificamente aqueles que foram bem manejados com acenocumarol ou fenprocumon. A varfarina tem sido o AVK mais amplamente estudado neste contexto, mas apesar dos possíveis problemas de disponibilidade regional e das discrepâncias farmacocinéticas, dados anteriores não sugeriram diferenças significativas no controle da anticoagulação, complicações hemorrágicas ou eventos tromboembólicos.^8 , 9^ Embora faltem ensaios clínicos comparativos no contexto da FA, num cenário que combina um controle estrito do índice internacional normalizado (INR) e a utilização de AVK a longo prazo, é pouco provável que o tipo de AVK tenha um grande impacto nos resultados.

A duração média da FA no FRAIL-AF foi de 12 a 13 anos, o que não é inesperado, uma vez que os pacientes foram considerados tolerantes a AVK. A tolerabilidade aos AVKs implica que os fatores de risco para sangramento seriam menos comuns entre a população randomizada no estudo. Apesar da elevada prevalência de polifarmácia, a variabilidade do INR foi atenuada, uma vez que os pacientes foram rigorosamente tratados por 8 serviços na Holanda especializados em doenças trombóticas. A hipertensão, outro fator de risco conhecido para sangramento, foi menos prevalente do que em ensaios anteriores (53%), assim como a terapia antiplaquetária concomitante (2,2%). Uma análise de subgrupo do estudo ARISTOLTE sugeriu que mesmo pacientes considerados experientes no uso de AVK se beneficiariam com a mudança para apixabana. No entanto, uma história de experiência com AVK foi definida como o recebimento de um AVK por pelo menos 30 dias em qualquer momento antes da inclusão.^10^ Da mesma forma, tanto nos estudos RE-LY quanto no ENGAGE-AF, o ponto de corte para a mesma definição de subgrupo foi de 60 dias.^11 , 12^ Um limiar de tempo tão curto pode não ter sido suficiente para selecionar adequadamente os pacientes que eram verdadeiramente tolerantes aos AVK. A Tabela 1 compara as características basais dos pacientes de ensaios NOAC anteriores e daqueles incluídos no FRAIL-AF.^13 , 14^

**Tabela 1:** Características basais dos pacientes de ensaios NOAC anteriores em fibrilação atrial e do ensaio FRAIL-AF^1 , 7 , 14^

Características base		FRAIL-AF	Ensaios NOAC anteriores			
			RE-LY	ROCKET-AF	ARISTOTLE	ENGAGE AF-TIMI 48
**Pacientes (n)**		1.323 *	18.113	14.264	18.201	21.105
**Tipo de NOAC**		Não especificado	Dabigatrana	Rivaroxabana	Apixabana	Edoxabana
**Variáveis de linha de base**					
	Idade, mediana, anos	82,9 †	71 †	73	70	72
	Idade ≥75 anos, (%)	100	28,1	43,2	31,2	40,0
	Sexo masculino, (%)	61,2	64	60	65	62
	FA paroxística, (%)	28	33	18	15	25
	Escore CHADS_2_, média	4,0 ‡	2,1	3,5	2,1	2,8
	Insuficiência cardíaca, (%)	21,1	32	63	35	57
	Hipertensão, (%)	53,0	79	91	87	94
	Diabetes, (%)	21,2	23	40	25	36
	Tromboembolismo prévio, (%)	19,3	20	55	19	28
	Uso anterior de aspirina, (%)	2,2	40	37	31	29
	Uso anterior de AVK, (%)	100	50	62	57	59
	História de sangramento clinicamente relevante, (%)	14,6 §	NI	NI	16,7 //	NI

FA: fibrilação atrial; NOAC: anticoagulante oral sem vitamina K; NI: não informado; AVK: antagonista da vitamina K;

* intenção de tratar a população;

† média;

‡ mediana do escore CHADS_2_Vasc_2_;

§ sangramento maior;

// sangramento clinicamente relevante ou espontâneo.

A população do estudo FRAIL-AF reflete com mais precisão pacientes idosos com FA com experiência no uso de AVK. Quando comparados com indivíduos definidos como experientes em AVK no estudo ARISTOTLE, os pacientes do FRAIL-AF tinham um histórico basal de menos eventos hemorrágicos (14,6% vs 21,7%).^1 , 10^ Além disso, a menor incidência de sangramentos maiores quando comparado aos pacientes com idade ≥75 anos em estudos anteriores, apoia ainda mais este conceito ( Tabela 2 ).^1 , 13^ Em uma coorte publicada anteriormente de 472 pacientes idosos consecutivos com FA tratados com varfarina, um aumento de 3 vezes no sangramento maior nos primeiros 90 dias de terapia foi relatada entre aqueles ≥80 anos. Embora o risco mais elevado tenha persistido ao longo do primeiro ano, a taxa de interrupção da terapia com varfarina atingiu o pico logo após o início do tratamento e permaneceu estável após os primeiros 6 meses de acompanhamento.^15^

**Tabela 2:** Resultados clínicos de ensaios anteriores de NOAC em fibrilação atrial e do ensaio FRAIL-AF^1 , 7 , 13 , 14^

Os resultados clínicos		FRAIL-AF	Ensaios NOAC anteriores			
			RE-LY	ROCKET -AF	ARISTOTLE	ENGAGE AF-TIMI 48
**Acompanhamento, mediana, anos**		0,94 *	2,0	1,9	1,8	2,8
**Resultados (braço tratado com AVK)**						
	Evento tromboembólico, (%/a)	2,1	1,7	2,2	1,6	1,8
	Total ≥75 anos	2,1	2,1	2,6	2,2	1,5
	Sangramento maior, (%/a)	2,6	3,4	3,4	3,1	3,4
	Total ≥75 anos	2,6	4,5	3,5	5,2	3,3
**Resultados (braço tratado com NOAC)**						
	Evento tromboembólico, (%/a)	2,6	1,1 †	1,7	1,3	1,6 ‡
	Total ≥75 anos	2,6	1,4 †	2,1	1,6	0,9 ‡
	Sangramento maior, (%/a)	3,9	3,1 †	3,6	2,1	2,8 ‡
	Total ≥75 anos	3,9	5,3 †	4,0	3,3	2,7 ‡

NOAC: anticoagulante oral sem vitamina K; AVK: antagonista da vitamina K; a: ano;

* média;

† dabigatrana 150 mg duas vezes ao dia;

‡ edoxabana 60 mg qd sangramento maior

Indivíduos com fatores de risco de HIC não controlados ou ocultos, como angiopatia amiloide subjacente, têm maior probabilidade de interromper a anticoagulação logo após o início do tratamento, diferindo assim do perfil de paciente definido no ensaio FRAIL-AF. Como tal, não foi inesperado que a maior taxa de eventos hemorrágicos no braço NOAC tenha sido associada principalmente a hemorragias CRNM de origem gastrointestinal e urogenital. À primeira vista, estes eventos podem parecer menos significativos, mas também representam um fardo substancial para os sistemas de saúde, pacientes e famílias, ao resultarem potencialmente em hospitalização, procedimentos médicos e ajustes adicionais de medicação para manejo terapêutico. Até 45% dos pacientes com sangramento CRNM associado à terapia anticoagulante oral podem necessitar de pequenas cirurgias ou procedimentos intervencionistas.^16^ No FRAIL-AF, possivelmente múltiplas interações medicamentosas imprevistas com subsequente farmacocinéticas imprevisíveis podem ter sido associadas ao maior número de eventos hemorrágicos naqueles que trocaram a terapia anticoagulante.

Sendo um ensaio pragmático, o estudo não foi desenhado para comparar desfechos de acordo com diferentes tipos de NOACs, e tais resultados devem ser considerados apenas como geradores de hipóteses. Os ensaios pragmáticos são concebidos para comparar estratégias de gestão do mundo real em amplos grupos de pacientes, refletindo principalmente aqueles que receberiam a intervenção na prática clínica num determinado cenário ou região. No entanto, como os tratamentos são frequentemente não cegos nestes estudos, muitos fatores de confusão podem ser fontes de vieses e afetarem diretamente os resultados.^17 , 18^ Isto é particularmente relevante no FRAIL-AF, uma vez que apenas os resultados fatais foram sistematicamente julgados por um comité independente e cego, e publicações anteriores sugeriram taxas de sangramento diferentes associadas a NOACs individuais. A apixabana foi particularmente relacionada a menos complicações hemorrágicas entre pacientes com FA, e a rivaroxabana foi o NOAC mais comumente usado no estudo FRAIL-AF.^19^ Finalmente, a adoção de clínicas especializadas em anticoagulação para controle do INR pode não estar disponível na maioria das circunstâncias em que os AVKs são prescritos. Apesar da tentativa dos autores de maximizar a qualidade do tratamento com AVK e abordar uma questão importante e relevante para a região onde o ensaio foi realizado, os resultados não devem ser extrapolados para situações em que o controle do INR possa ser menos eficiente.

Reconhecer a singularidade do FRAIL-AF é essencial para apreciar os seus pontos fortes e limitações. Embora o ensaio tenha fornecido uma contribuição significativa para este tópico, a combinação de pacientes frágeis altamente selecionados com FA tratados dentro de uma estrutura de saúde específica limita a validade externa do estudo quando se consideram cenários fora do sistema de saúde holandês. Os médicos não devem generalizar os resultados para circunstâncias clínicas divergentes, tais como a seleção do anticoagulante oral mais apropriado ao iniciar o tratamento para um indivíduo frágil que nunca recebeu AVK. É essencial estar ciente de que esta questão não foi abordada pelo FRAIL-AF e exigiria um ensaio clínico distinto que ainda não foi realizado. No entanto, numa coorte recentemente publicada de mais de 650 mil idosos beneficiários do Medicare com FA que iniciaram terapêutica anticoagulante oral, a apixabana foi associada a resultados de eficácia e segurança significativamente melhores quando comparado com a varfarina. Os resultados foram consistentes em vários níveis de fragilidade, enquanto apenas os pacientes não frágeis tiveram melhor desempenho com dabigatrana e rivaroxabana.^20^ Desta forma, as recomendações atuais ainda favorecem os NOACs para esta indicação, com base em dados derivados de subgrupos de pacientes idosos e estudos observacionais de grande escala.^4 , 21^

## Conclusão

O estudo de Joosten et al. lembrou aos cardiologistas que a MBE ainda é o método mais eficaz para responder a uma questão científica. Os dados obtidos de aproximadamente 72 mil pacientes avaliados em ensaios anteriores de NOACs foram inestimáveis para a prática clínica. Contudo, as restantes áreas de incerteza onde tais resultados não se aplicam não devem ser ignoradas. Reconhecer os limites do conhecimento médico atual é essencial para criar oportunidades para novos progressos. Independentemente das suas limitações, a maior força do FRAIL-AF é ratificar que mesmo conceitos aparentemente bem definidos devem ser submetidos ao escrutínio do método científico e da MBE. O ônus da prova na demonstração da segurança da troca da anticoagulação oral em pacientes frágeis com FA em terapia estável com AVK permanece indefinido.
