# Supplementary material for: Fragilidade e Fibrilação Atrial: Um Olhar Mais Atento ao Estudo FRAIL-AF
Source: Arq Bras Cardiol. 2024 Jun 3;121(5):e20230671. [Article in Portuguese] doi: 10.36660/abc.20230671 (PMC11164436; doi:10.36660/abc.20230671)
Supplement: Translation

# Translation: Frailty and Atrial Fibrillation: A Closer Look at the FRAIL-AF Trial

**DOI:** 10.36660/abc.20230671i

**Authors:** Roberto Muniz Ferreira

**Affiliations:** 1 Rio de Janeiro RJ Brazil Universidade Federal do Rio de Janeiro, Instituto do Coração Edson Saad, Rio de Janeiro, RJ – Brazil; 2 Rio de Janeiro RJ Brazil Hospital Samaritano – Departamento de Cardiologia, Rio de Janeiro, RJ – Brazil

**Keywords:** Atrial Fibrillation, Anticoagulants, Evidence-Based Medicine, Frail Elderly

## Introduction

Although evidence-based medicine (EBM) has contributed to establishing the foundations of patient care for over 40 years, scientific principles are still often disregarded in clinical decision-making. As a result, EBM may be mistakenly characterized as a barrier that limits the applicability of poorly recognized concepts, otherwise perceived as thoroughly supported by medical literature. The critical analysis of medical publications should not rely on methods estranged from the scientific realm, and EBM provides the means to limit such hazardous misconceptions.

Despite the continuous progress in medical knowledge, numerous areas of uncertainty persist in clinical practice. In such cases, decision-making typically depends on information originating from dissimilar scenarios, unsuitably designed research or personal experience, and alternatives that are inherently fragile as supporting evidence. Indeed, frailty is a problem that most physicians ultimately have to manage, whether referring to scientific evidence or patient profiles. Greater difficulties arise when both are a source of fragility since misjudged decisions tend to have even poorer outcomes. Atrial fibrillation (AF) is a topic where this association potentially occurs.

## The FRAIL-AF Trial

The recently published FRAIL-AF study (Safety of Switching from a Vitamin K Antagonist to a Non-Vitamin K Antagonist Oral Anticoagulant in Frail Older Patients with AF) was a pragmatic, open-label, randomized superiority trial that assessed whether frail patients with AF adequately managed with vitamin K antagonists (VKAs) should be switched to a non-vitamin K oral anticoagulant (NOAC). The main inclusion criteria consisted of patients aged ≥75 years with a Groningen Frailty Indicator (GFI) score ≥3, whereas those with valvular AF or severe renal dysfunction were excluded. The primary outcome was the first occurrence of major or clinically relevant non-major bleeding over 12 months of follow-up.^1^

Between January 2018 and June 2022, 1,330 patients were randomized. The mean age was 83 years, the median GFI was 4 and the majority were males (61.2%). In the NOAC group, rivaroxaban was most frequently prescribed (50.2%), followed by apixaban (17.4%) and edoxaban (16.5%). After a mean follow-up of 344 days and 163 primary outcome events, the previously defined protocol required the trial's interruption for futility, though the NOAC group was associated with a significant 69% increase in bleeding rates (HR 1.69; 95% CI 1.23-2.32; p=0.00112). There were no differences in thromboembolic events or deaths.^1^

Based on the available data from multiple randomized trials and meta-analyses demonstrating an improved safety profile with NOACs when compared to VKAs, these drugs have been established as the primary treatment regimen for the prevention of thromboembolic events for most patients with AF. Much of the benefit is derived from significant reductions in major bleeding, particularly intracranial hemorrhage (ICH).^2 , 3^Accordingly, the European Rhythm Association (ERA) suggests that anticoagulation should not be withheld in older individuals with AF purely based on age and that NOACs are also the preferred option in this population.^3^

However, the recently published practical guide on NOAC use developed by the ERA also recognized that frailty characterizes a unique group of patients where the indication of any oral anticoagulant still represents an area of uncertainty.^3^In this regard, Joosten et al.^1^should be commended for attempting to answer one of the concerns not appropriately addressed in previous large-scale NOAC trials.^1 , 4^Frailty is a syndrome that extends beyond advanced age and involves multiple integrating factors, such as polypharmacy and severe comorbidities, which ultimately impose a high level of dependability and vulnerability. In the aforementioned publication, the ERA provided valuable insights into the importance of a comprehensive geriatric assessment before anticoagulation is considered in this context. Although the authors suggested the Canadian Study of Health and Aging (CHSA) Clinical Frailty Scale for this purpose, other validated scales could also be employed, such as the GFI which was used in FRAIL-AF.^3 , 5 , 6^

The ERA has recommended that individuals with AF classified as severely frail by the CHSA scale typically should not receive anticoagulation. Such patients would also be defined as having a comparable GFI score of 3 (capable of limited self-care, confined to bed or chair and about <50% of waking hours) or 4 (completely disabled, cannot carry on any self-care, totally confined to bed or chair).^5 , 6^Most importantly, over 74% of patients in FRAIL-AF had a GFI score of 4, and therefore would generally not be considered for anticoagulation in most clinical scenarios, as proposed by the ERA.^1 , 3^Such a distinctive patient profile underscores one of the main differences between previous trials and FRAIL-AF.

A fundamental reason for the systematic exclusion of frail individuals from previous studies is the poor short-term survival rate associated with the condition. Most AF trials considered minimum life expectancy thresholds of 1 to 2 years for enrolment, thus limiting the number of randomized frail patients.^4^In FRAIL-AF, the estimated annual mortality rate in the VKA group was 7.4%, which was approximately 25% greater than the subgroup of patients ≥75 years treated with warfarin (5.97%) in the ARISTOTLE trial.^1 , 7^This difference in survival rates emphasizes the independent effect frailty has on prognosis, even among elderly individuals.

In addition, FRAIL-AF analyzed a particular subgroup of frail patients with AF, specifically those who were well-managed with either acenocoumarol or phenprocoumon. Warfarin has been the most widely studied VKA in this context, but despite potential in-class regional availability issues and pharmacokinetic discrepancies, previous data have not suggested significant differences in anticoagulation control, bleeding complications, or thromboembolic events.^8 , 9^Though head-to-head clinical trials in AF are lacking, in a setting combining strict international normalized ratio (INR) control and long-term VKA usage, the type of VKA would be unlikely to have a major impact on the results.

Mean AF duration in FRAIL-AF was 12 to 13 years, which is not unanticipated given patients were considered VKA-tolerant. Tolerability to VKAs implies that risk factors for bleeding would expectedly be less common among the randomized population in the study. Despite the high prevalence of polypharmacy, INR variability was attenuated, since patients were rigorously managed by 8 specialized Dutch thrombosis services. Hypertension, another known risk factor for bleeding, was less prevalent than in previous trials (53%), as was concomitant antiplatelet therapy (2.2%). A subgroup analysis of the ARISTOLTE trial suggested that even VKA-experienced patients would benefit from switching to apixaban. However, a history of VKA experience was defined as receiving a VKA for at least 30 days at any time before enrolment.^10^Similarly, in both the RE-LY and ENGAGE-AF trials the cut-off for the same subgroup definition was 60 days.^11 , 12^Such a short time threshold may not have been sufficient to adequately select patients who were truly VKA tolerant. Table 1 compares baseline patient characteristics from previous NOAC trials and those included in FRAIL-AF.^13 , 14^

**Table 1:** Baseline patient characteristics from previous NOAC trials in atrial fibrillation and the FRAIL-AF trial^1 , 7 , 14^

Baseline characteristics		FRAIL-AF	Previous NOAC trials			
			RE-LY	ROCKET-AF	ARISTOTLE	ENGAGE AF-TIMI 48
**Patients (n)**		1,323 *	18,113	14,264	18,201	21,105
**Type of NOAC**		Not specified	Dabigatran	Rivaroxaban	Apixaban	Edoxaban
**Baseline variables**					
	Age, median, years	82.9 †	71 †	73	70	72
	Age ≥75 years, (%)	100	28.1	43.2	31.2	40.0
	Male sex, (%)	61.2	64	60	65	62
	Paroxysmal AF, (%)	28	33	18	15	25
	CHADS2 score, mean	4.0 ‡	2.1	3.5	2.1	2.8
	Heart failure, (%)	21.1	32	63	35	57
	Hypertension, (%)	53.0	79	91	87	94
	Diabetes, (%)	21.2	23	40	25	36
	Previous thromboembolism, (%)	19.3	20	55	19	28
	Previous aspirin use, (%)	2.2	40	37	31	29
	Previous VKA use, (%)	100	50	62	57	59
	History of clinically relevant bleeding, (%)	14.6 §	NI	NI	16.7 //	NI

AF: atrial fibrillation; NOAC: non-vitamin K oral anticoagulant; NR: not reported; VKA: vitamin K antagonist;

* intention to treat population;

† mean;

‡ median CHADS_2_Vasc_2_score;

§ major bleeding;

// clinically relevant or spontaneous bleeding.

The FRAIL-AF trial population more accurately reflects VKA-experienced elderly patients with AF. When compared to individuals defined as VKA-experienced in the ARISTOTLE trial, patients in FRAIL-AF had a baseline history of fewer bleeding events (14.6% vs 21.7%).^1 , 10^Furthermore, the lower incidence of major bleeding when compared to patients aged ≥75 years from previous trials, further supports this concept ( Table 2 ).^1 , 13^In a previously published cohort of 472 consecutive elderly patients with AF treated with warfarin, a 3-fold increase in major bleeding in the first 90 days of therapy was reported among those ≥80 years. Although the higher risk persisted throughout the first year, the interruption rate of warfarin therapy peaked early after treatment initiation and remained stable after the first 6 months of follow-up.^15^

**Table 2:** Clinical outcomes from previous NOAC trials in atrial fibrillation and the FRAIL-AF trial^1 , 7 , 13 , 14^

Clinical outcomes		FRAIL-AF	Previous NOAC trials			
			RE-LY	ROCKET -AF	ARISTOTLE	ENGAGE AF-TIMI 48
**Follow-up, median, years**		0.94 *	2.0	1.9	1.8	2.8
**Outcomes (VKA-treated arm)**						
	Thromboembolic event, (%/y)	2.1	1.7	2.2	1.6	1.8
	Total ≥75 years	2.1	2.1	2.6	2.2	1.5
	Major bleeding, (%/y)	2.6	3.4	3.4	3.1	3.4
	Total ≥75 years	2.6	4.5	3.5	5.2	3.3
**Outcomes (NOAC-treated arm)**						
	Thromboembolic event, (%/y)	2.6	1.1 †	1.7	1.3	1.6 ‡
	Total ≥75 years	2.6	1.4 †	2.1	1.6	0.9 ‡
	Major bleeding, (%/y)	3.9	3.1 †	3.6	2.1	2.8 ‡
	Total ≥75 years	3.9	5.3 †	4.0	3.3	2.7 ‡

NOAC: non-vitamin K oral anticoagulant; VKA: vitamin K antagonist; y: year;

* mean;

† dabigatran 150mg bid;

‡ edoxaban 60mg qd major bleeding.

Individuals with uncontrolled or occult ICH risk factors, such as underlying amyloid angiopathy, are more likely to stop anticoagulation shortly after treatment initiation, thus differing from the patient profile defined in the FRAIL-AF trial. As such, it was not unexpected that the higher rate of bleeding events in the NOAC arm was mostly due to clinically relevant non-major (CRNM) bleeding from gastrointestinal and urogenital sources. At first, these events may seem less significant, but they also convey a substantial burden to healthcare systems, patients, and families, by potentially resulting in hospitalization, medical procedures, and further medication adjustments for therapeutic management. Up to 45% of patients with CRNM bleeding associated with oral anticoagulant therapy may require minor surgery or interventional procedures.^16^In FRAIL-AF, possibly multiple unforeseen drug interactions and subsequent unpredictable pharmacokinetics may have been associated with the greater number of bleeding events in those who switched anticoagulation therapy.

As a pragmatic trial, the study was not designed to compare outcomes according to different types of NOACs, and such results should only be regarded as hypothesis-generating. Pragmatic trials are designed to compare real-world management strategies in broad patient groups, primarily reflecting those who would receive the intervention in clinical practice within a given scenario or region. However, as treatments are frequently unblinded in these studies, many confounders may be a source of bias and directly affect the results.^17 , 18^This is particularly relevant in FRAIL-AF, since only fatal outcomes were systematically adjudicated by an independent blinded committee, and previous publications have suggested dissimilar bleeding rates associated with individual NOACs. Apixaban has particularly been related to fewer bleeding complications among patients with AF, and rivaroxaban was the most commonly used NOAC in the FRAIL-AF trial.^19^Finally, the adoption of specialized anticoagulation clinics for INR control may not be available in most circumstances where VKAs are prescribed. Despite the authors’ attempt to maximize the quality of VKA treatment and address an important issue relevant to the region where the trial was undertaken, the results should not be extrapolated to situations where INR control may be less efficient.

Recognizing the uniqueness of FRAIL-AF is essential to appreciate its strengths and limitations. Though the trial has provided a significant contribution to this topic, the combination of highly selected frail patients with AF managed within a specific healthcare structure limits the external validity of the study when considering scenarios outside of the Dutch healthcare system. Physicians should not generalize the results to divergent clinical circumstances, such as selecting the most appropriate oral anticoagulant when initiating treatment for a VKA-naive frail individual. It is essential to be aware that this issue was not addressed by FRAIL-AF, and would require a distinct explanatory trial that has yet to be undertaken. Nevertheless, in a recently published cohort of over 650 thousand elderly Medicare beneficiaries with AF who were initiated on oral anticoagulant therapy, apixaban was associated with significantly improved efficacy and safety outcomes when compared to warfarin. The results were consistent throughout various levels of frailty, whereas only non-frail patients fared better with dabigatran and rivaroxaban.^20^As such, current recommendations still favor NOACs for this indication, based on data derived from elderly patient subgroups and large-scale observational studies.^4 , 21^

## Conclusion

The trial by Joosten et al. has reminded cardiologists that EBM is still the most effective method to answer a scientific question. The data obtained from approximately 72 thousand patients evaluated in previous NOAC trials has been invaluable for clinical practice. However, the remaining areas of uncertainty where such results do not apply should not be overlooked. Acknowledging the limits of current medical knowledge is essential to create the opportunity for further progress. Regardless of its limitations, the greatest strength of FRAIL-AF is ratifying that even apparently well-defined concepts should be subject to scrutiny by the scientific method and EBM. The burden of proof in demonstrating the safety of switching oral anticoagulation in frail AF patients on stable VKA therapy remains unsettled.
